# Microglia stabilize sleep homeostasis via adenosine A_3_ receptor signaling

**DOI:** 10.1126/sciadv.aef3957

**Published:** 2026-09-11

**Authors:** Zhong Zhao, Xiaochun Gu, Jing Yu, Xueli Chen, Lifeng Zhang, Ting Zhao, Wei Ye, Heping Cheng

**Affiliations:** ^1^ PKU-Nanjing Institute of Translational Medicine, Nanjing 210032, China.; ^2^ National Platform for Medical Engineering Education Integration, Southeast University, Nanjing 211899, China.; ^3^ Nurturing Center of Jiangsu Province for State Laboratory of AI Imaging & Interventional Radiology, Department of Radiology, Zhongda Hospital, Medical School, Southeast University, Nanjing 210009, China.; ^4^ Key Laboratory of Developmental Genes and Human Diseases, Department of Histology Embryology, Medical School, Southeast University, Nanjing 210009, China.; ^5^ Nanjing Raygenitm Biotech Co. Ltd., Nanjing 210032, China.; ^6^ National Biomedical Imaging Center, Beijing 100871, China.; ^7^ State Key Laboratory of Membrane Biology, Beijing Key Laboratory of Cardiometabolic Molecular Medicine, Peking-Tsinghua Center for Life Sciences, Institute of Molecular Medicine, College of Future Technology, Peking University, Beijing 100871, China.

## Abstract

Sleep homeostasis maintains the sleep-wake balance through sleep pressure, a process partly orchestrated by the accumulation of extracellular adenosine (eADO). Microglial Ca^2+^ activity has been implicated in sleep regulation, but the mechanism whereby microglia sense sleep pressure remains unclear. Here, we show that microglia regulate sleep homeostasis through brain state–dependent calcium ion activity driven by adenosine A_3_ receptor (A_3_R) signaling. Using miniaturized two-photon microscopy in freely behaving mice, we demonstrate that microglial calcium ion activity is rapidly altered by brain-state transitions. Pharmacological experiments reveal that microglial calcium ion dynamics are predominantly mediated by A_3_R in response to brain state–dependent eADO oscillations. Microglia-specific deletion of A_3_R attenuates these state-dependent calcium ion dynamics, impairs microglial morphological plasticity across sleep-wake cycles, and leads to sleep fragmentation by increasing transitions between wakefulness and non-rapid eye movement (NREM) sleep. Together, these findings establish that microglia contribute to sleep homeostasis by stabilizing both wakefulness and NREM sleep, a process partly involving eADO-A_3_R signaling.

## INTRODUCTION

Sleep homeostasis, which is driven by sleep pressure, orchestrates the balance between sleep and wakefulness (*1*–*3*). At the molecular level, it involves endogenous sleep-promoting substances [such as adenosine, tumor necrosis factor–α (TNF-α), and interleukin-1β], whose concentrations dynamically fluctuate with sleep pressure and contribute to the regulation of sleep (*4*–*6*). Among these, extracellular adenosine (eADO) is a key molecule that progressively accumulates during wakefulness and drives sleep initiation (*4*, *5*).

eADO exerts its physiological effects through four G protein–coupled adenosine receptor subtypes: A_1_R, A_2A_R, A_2B_R, and A_3_R (*7*). Among them, A_1_R and A_2A_R have been shown to modulate sleep in a brain region–specific manner (*8*, *9*), whereas A_2B_R, a low-affinity receptor (*10*), primarily responds to elevated eADO under pathological conditions (*11*). In contrast, the physiological role of A_3_R in sleep regulation has not yet been clarified.

Microglia, the resident immune cells of the central nervous system (CNS), are pivotal in regulating neural activity, synaptic pruning, and neuroimmunity (*12*, *13*). Accumulating studies have established microglia as key modulators of sleep-wake states (*14*–*18*), yet the cellular mechanisms underlying their responses to sleep pressure and subsequent homeostatic regulation remain to be elucidated. In their surveillance state, microglia continuously extend and retract their highly dynamic processes to monitor neural activity and maintain CNS homeostasis (*14*, *19*–*21*). Their dynamics are regulated by the purinergic signaling (*22*). Through this pathway, P_2_Y_12_ receptors respond to adenosine 5′-triphosphate (ATP)/adenosine 5′-diphosphate (ADP) released from injured sites or neurons by inducing microglial process extension and morphological changes (*23*–*25*). In this process, adenosine-activated A_3_R promotes ADP-induced extension of microglial processes (*26*). However, whether microglial A_3_R participates in physiological sleep regulation and how adenosine signaling influences microglial dynamics across natural sleep-wake cycles remain unknown.

Miniaturized two-photon microscopy (mTPM) has provided a transformative tool for in vivo imaging in freely behaving mice (*27*, *28*), as evidenced by brain state–dependent microglial surveillance (*14*). Specifically, microglial surveillance undergoes rapid and robust remodeling across natural sleep-wake cycles and in response to sleep deprivation stress through high-resolution time-lapse in vivo imaging in freely behaving mice (*14*). Further, norepinephrine (NE) signaling modulates microglial surveillance, thereby serving as a key mechanism coupling microglia to brain state (*14*). In the present study, we apply this powerful in vivo imaging approach to investigating the microglial signaling involved in regulating sleep homeostasis.

We aim to conduct long-term in vivo imaging of cortical microglial Ca^2+^ activity, morphology, and eADO levels in freely behaving mice using mTPM. By classifying brain states based on simultaneously recorded electroencephalogram (EEG) and electromyogram (EMG) signals, we intend to examine the relationship between sleep-wake states, eADO oscillations, and microglial Ca^2+^ activity. Particular attention will be paid to elucidate the role of microglial A_3_R signaling as eADO-responsive elements. Together, these approaches will specify the role and mechanism of microglia as active modulators of sleep homeostasis.

## RESULTS

### Microglial Ca^2+^ activity during natural sleep-wake cycles

**Fig. 1.**
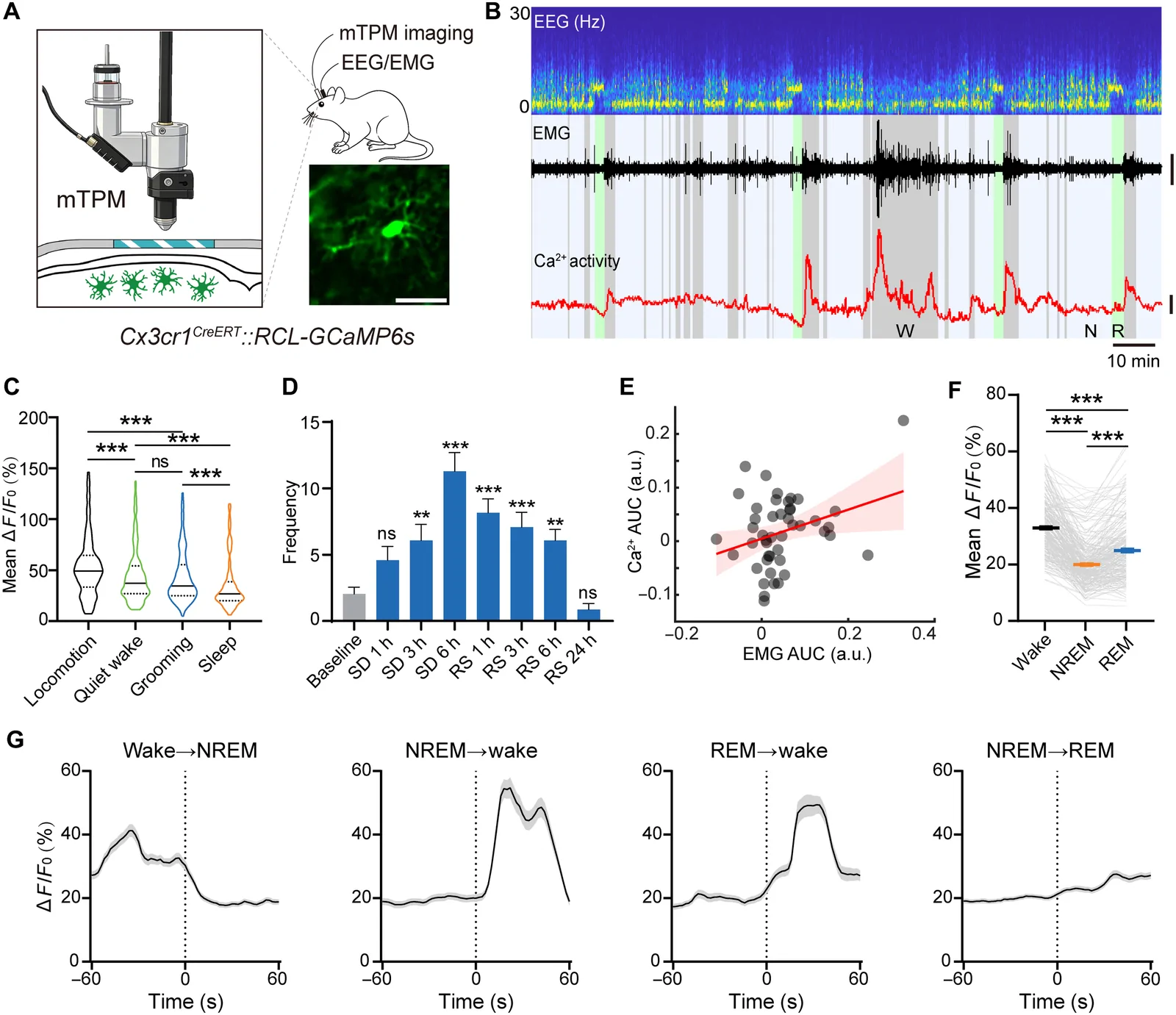
Brain state–dependent microglial Ca^2+^ activity. (**A**) Experimental setup: mTPM and EEG/EMG recordings in freely behaving mice with tamoxifen-induced expression of GCaMP6s in microglia. A representative mTPM image of microglia in a *Cx3cr1^CreERT^::RCL-GCaMP6s* mouse. Scale bar, 30 μm. (**B**) Representative long-term microglial Ca^2+^ activity during sleep-wake cycles in freely behaving mice, showing EEG (0 to 30 Hz spectrogram), EMG (scale bar, 100 μV), and *z*-scored Ca^2+^ signals from a microglial cell (scale bar, 2 *z*-score). Brain states are color coded: wake (W; gray), NREM (N; light gray), and REM (R; green). (**C**) Mean microglial Ca^2+^ activity under different behavioral conditions. Friedman test with Dunn’s post hoc test was used. *n* = 166 cells from six sessions of four mice. (**D**) The number of spontaneous Ca^2+^ transients in microglia recorded over a 15-min period during sleep deprivation (SD) and recovery sleep (RS). *n* = 30, 42, 40, 35, 54, 47, 55, and 16 cells from three mice. Unpaired *t* test. h, hours. (**E**) Correlation between Ca^2+^ AUC and EMG AUC. Each circle represents one event (*n* = 46 from four mice). Red line, linear regression fit; shaded area, 95% confidence interval (CI). Pearson’s *r* = 0.32 and *P* < 0.05. The nonsignificant correlation (*P* > 0.05) confirms specificity. a.u., arbitrary units. (**F**) Mean microglial Ca^2+^ activity during different brain states. Friedman test with Dunn’s post hoc test was used for paired measures. The gray solid line represents Ca^2+^ activity of the same cell across the three brain states. *n* = 220 cells from 19 sessions of six mice. **P < 0.01 and ***P < 0.001. ns, not significant. (**G**) Microglial Ca^2+^ activity during brain state transitions. The vertical dotted line represents the transition time. *n* = 143, 79, 61, and 164 cells from 10 sessions of five mice. Data are shown as means ± SEM.

To investigate how cortical microglial Ca^2+^ activity changes during natural sleep-wake cycles, we adopted a recently developed mTPM system (FHIRM-TPM) from our laboratory for long-term in vivo imaging in freely behaving mice (*27*, *28*). Applying this system to a transgenic mouse model for microglia-specific GCaMP6s expression (*Cx3cr1^CreERT^::RCL-GCaMP6s*) (fig. S1A), we performed continuous in vivo imaging in the somatosensory cortex (Fig. 1A). By positioning the focal plane 50 to 100 μm beneath the cortical surface, we were able to stably record microglial Ca^2+^ dynamics at a high frame rate (5 Hz) over periods exceeding 6 hours. Throughout these prolonged recordings, the animals exhibited natural behavior and undisturbed sleep-wake cycles. To correlate the imaging data with brain states, we simultaneously recorded EEG/EMG signals and behavioral video for precise classification (Fig. 1A). Similar to those reported in the literature (*29*–*31*), our multimodal imaging system also revealed the typical temporal distribution pattern of brain states in mice, characterized by a large number of very short-duration episodes and a smaller number of long-duration episodes across wakefulness, non-rapid eye movement (NREM) sleep, and rapid eye movement (REM) sleep (fig. S1B).

Microglial Ca^2+^ activity in freely behaving mice was enhanced during wakefulness (Fig. 1B). Furthermore, this activity was finely tuned by behavior, peaking during locomotion and dropping to substantially lower levels during quiet wakefulness and grooming, decreasing further during sleep, namely, NREM sleep and REM sleep (Fig. 1C and movie S1). At the onset of locomotion, microglial Ca^2+^ activity increased rapidly, whereas it declined precipitously when mice transitioned to quiet wakefulness or sleep (fig. S2A). In addition, grooming behavior was accompanied by a marked elevation in microglial Ca^2+^ activity (fig. S2A). Beyond its behavioral patterns, the frequency of spontaneous Ca^2+^ transients was also modulated by sleep pressure, increasing with prolonged sleep deprivation and gradually returning to baseline during subsequent recovery sleep (Fig. 1D). Consistently, the size of microglial Ca^2+^ event correlated with the size of the corresponding EMG signal (Fig. 1E; Pearson’s *r* = 0.32, *P* < 0.05), whereas microglial Ca^2+^ activity showed no significant correlation with EEG delta, theta, sigma, or gamma power (fig. S2B). Analysis across sleep-wake states revealed that microglial Ca^2+^ activity was highest during wakefulness, intermediate in REM sleep, and lowest in NREM sleep (Fig. 1F). Compared with REM sleep, both wakefulness and NREM sleep exhibited larger rising and decay phases of microglial Ca^2+^ events; moreover, the amplitude of microglial Ca^2+^ events during wakefulness was higher than that during NREM sleep (fig. S2C). Notably, microglial Ca^2+^ activity shifted abruptly at transitions between wakefulness and NREM sleep, as well as from REM sleep to wakefulness, whereas the increase from NREM to REM sleep was only modest (Fig. 1G). Together, these results suggest that microglial Ca^2+^ activity exhibits robust, state-dependent dynamics during sleep-wake cycles, providing a foundation to investigate its functional role in the regulation of sleep homeostasis.

### Cortical eADO levels during natural sleep-wake cycles

**Fig. 2.**
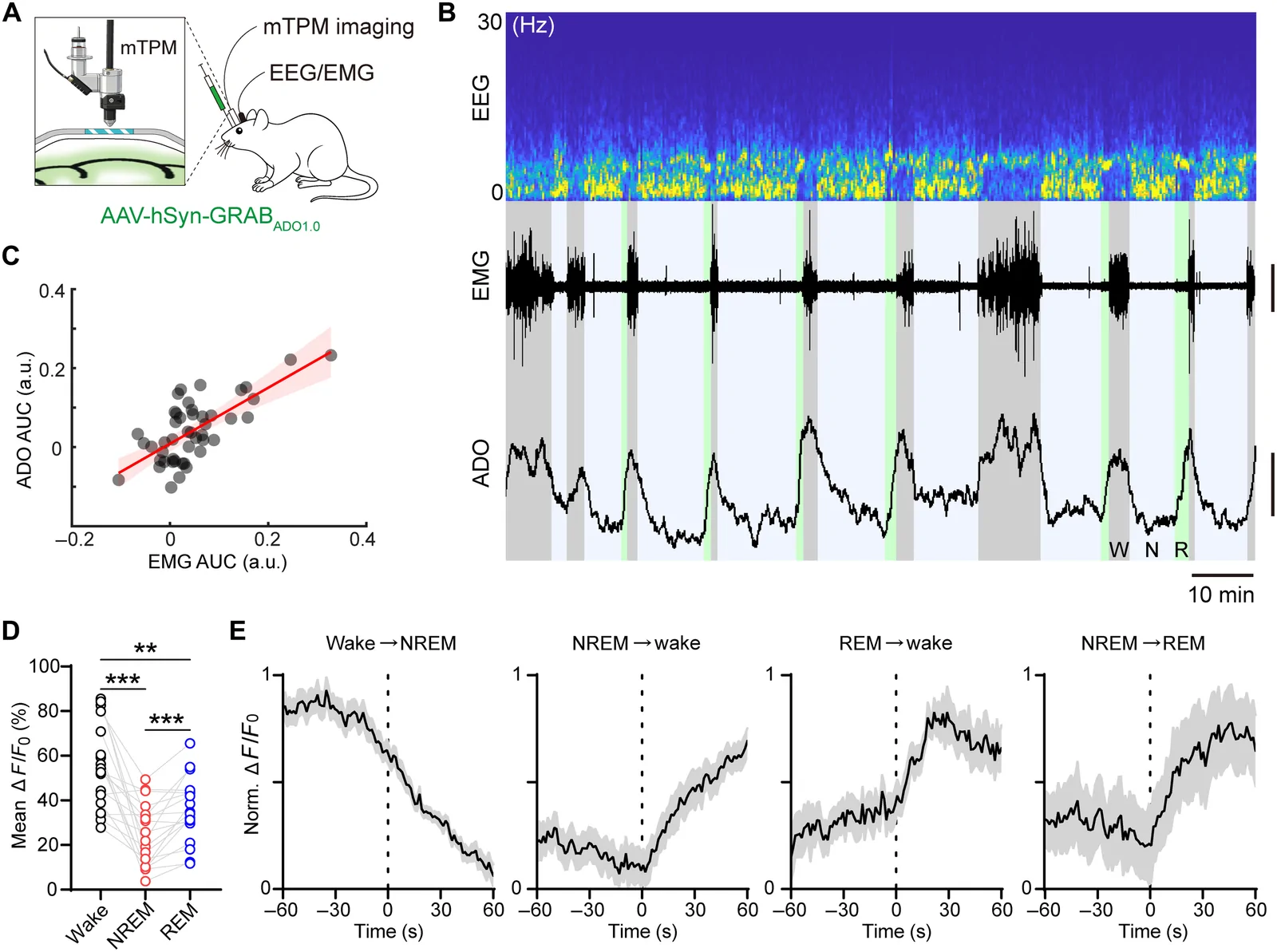
eADO oscillations during natural sleep-wake cycles. (**A**) Schematic diagram depicting mTPM images of eADO reported by the GRAB_ADO1.0_ sensor expressed in neurons. Freely behaving mice were head mounted with an mTPM and EEG/EMG electrodes. (**B**) Representative simultaneous recordings from somatosensory cortex during the sleep-wake cycles in freely behaving mice, showing EEG (0- to 30-Hz spectrogram), EMG (scale bar, 1 mV), and eADO (scale bar, 2 *z*-score) signals. (**C**) Correlation between ADO AUC and EMG AUC. Each circle represents one event (*n* = 46 from four mice). Red line, linear regression fit; shaded area, 95% CI. Pearson’s *r* = 0.7 and *P* < 0.0001. The nonsignificant correlation (*P* > 0.05) confirms specificity. (**D**) Mean eADO levels in different brain states. Data from the same recording are connected by lines. One-way analysis of variance (ANOVA) with Tukey’s post hoc test was used. *n* = 18 sessions from five mice. **P < 0.01 and ***P < 0.001. (**E**) Signal of the GRAB_ADO_ sensor during brain state transitions. The vertical dashed line represents the transition time. *n* = 30, 30, 13, and 17 events from four mice. Data are shown as means ± SEM.

The accumulation of eADO is a crucial factor driving sleep. In the cortex, eADO increases during sleep deprivation and gradually decreases during recovery sleep (*32*), linking its dynamics to sleep homeostasis. To investigate how cortical eADO fluctuates across brain states, we expressed the adenosine sensor AAV9-hSyn-GRAB_ADO1.0_ in the somatosensory cortex and performed long-term in vivo imaging using mTPM in freely behaving mice, while simultaneously monitoring brain states via EEG/EMG recordings (Fig. 2A).

We found that eADO displayed sustained high levels during wakefulness, declined to its lowest levels in NREM sleep, and was marked by brief, rapid increases during REM sleep (Fig. 2B). The eADO signal was positively correlated with EMG signal (Fig. 2C; Pearson’s *r* = 0.7, *P* < 0.0001). Quantification confirmed that eADO levels were highest during wakefulness, intermediate during REM sleep, and lowest during NREM sleep (Fig. 2D). Furthermore, eADO responded rapidly to brain state transitions, decreasing during the transition from wakefulness to NREM sleep but increasing sharply during transitions from NREM sleep to either wakefulness or REM sleep and again from REM sleep to wakefulness (Fig. 2E). Together, these results indicate that cortical eADO dynamics are closely coupled to sleep-wake transitions.

### eADO-A_3_R regulates microglial Ca^2+^ activity

**Fig. 3.**
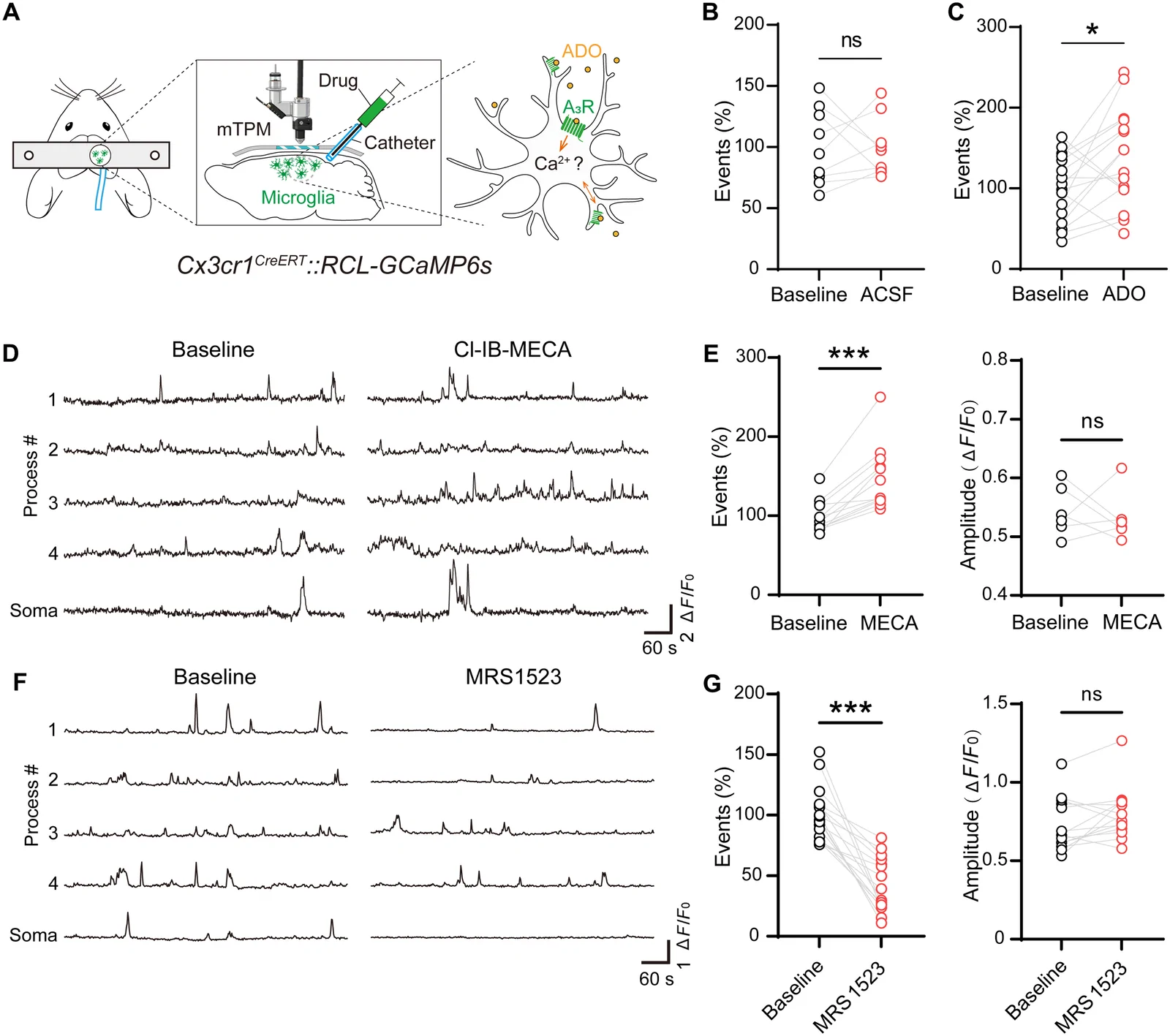
Microglial Ca^2+^ activity is modulated by eADO and A_3_R. (**A**) Schematic diagram depicting mTPM images of microglial Ca^2+^ activity in freely behaving mice. A drug infusion catheter was implanted in the subarachnoid space adjacent to the cranial window. Microglia were imaged following local application of ADO or A_3_R ant/agonists for 30 min. (**B** and **C**) The number of Ca^2+^ events within the FOV in 5-min recordings, 30 min after ACSF (B) or ADO (C) administration. Data from the same FOV are connected by lines. *n* = 9 FOVs from three mice (ACSF). *n* = 16 FOVs from five mice (ADO). Two-tailed paired *t* test. (**D** and **F**) Representative microglial Ca^2+^ signal in 10-min recordings, 30 min after Cl-IB-MECA (D) or MRS1523 (F) administration. (**E** and **G**) The number of Ca^2+^ events and amplitude in microglial processes in 5-min recordings, 30 min after Cl-IB-MECA (E) or MRS1523 (G) administration. Data from the same FOV are connected by lines. *n* = 12 [(E), event], 6 [(E), amplitude], 17 [(G), event], and 14 [(G), amplitude] FOVs from three mice. Wilcoxon test [(E) and (G)]. *P < 0.05 and ***P < 0.001.

Adenosine regulates Ca^2+^ activity through multiple adenosine receptors, with A_3_R being the expressed adenosine receptor in microglia (*22*, *26*). To investigate whether microglial Ca^2+^ activity is mediated by the eADO-A_3_R axis, we performed intracerebral administration of adenosine, an A_3_R agonist (Cl-IB-MECA), or an A_3_R antagonist (MRS1523) and assessed their effects on microglial Ca^2+^ activity (Fig. 3A). Given that drug administration might alter microglial motility, Ca^2+^ events were quantified using Astrocyte Quantitative Analysis (AQuA) software to minimize movement-related artifacts. Intracerebral administration of artificial cerebrospinal fluid (ACSF) did not change Ca^2+^ activity (Fig. 3B and fig. S3A), allowing us to track the dynamic pharmacological effects within the same field of view (FOV). Both adenosine and Cl-IB-MECA increased the number of Ca^2+^ events (Fig. 3, C to E), whereas MRS1523 decreased event frequency (Fig. 3, F and G). Neither Cl-IB-MECA nor MRS1523 altered the amplitude of microglial Ca^2+^ activity (Fig. 3, E and G). Furthermore, agonists and antagonists of A_1_R, A_2A_R, or A_2B_R had no effect on microglial Ca^2+^ activity (fig. S3, B and C).

To test whether these effects were directly mediated by microglial A_3_R, we generated microglia-specific A_3_R conditional knockout (cKO) mice by crossing *Adora3^fl/fl^* mice with *Cx3cr1^CreERT^* mice (fig. S4A). Tamoxifen was administered to 7-week-old mice to ablate *Adora3* in microglia. Quantitative reverse transcription polymerase chain reaction (PCR) analysis of isolated CD11b^+^/CD45^low^ microglia showed a ∼75% reduction in *Adora3* mRNA in A_3_R cKO mice, with no significant change in the negative (CD11b^−^/CD45^−^) cell population (fig. S4, B and C). The regulatory effects of Cl-IB-MECA and MRS1523 were significantly attenuated in these mice (fig. S4, D and E), indicating that microglial A_3_R directly regulates Ca^2+^ activity. Collectively, these findings establish the eADO-A_3_R pathway as a crucial regulator of microglial Ca^2+^ activity, enabling microglia to respond dynamically to eADO fluctuations across brain states.

### Microglial A_3_R deficiency fragmentizes wakefulness and NREM sleep

**Fig. 4.**
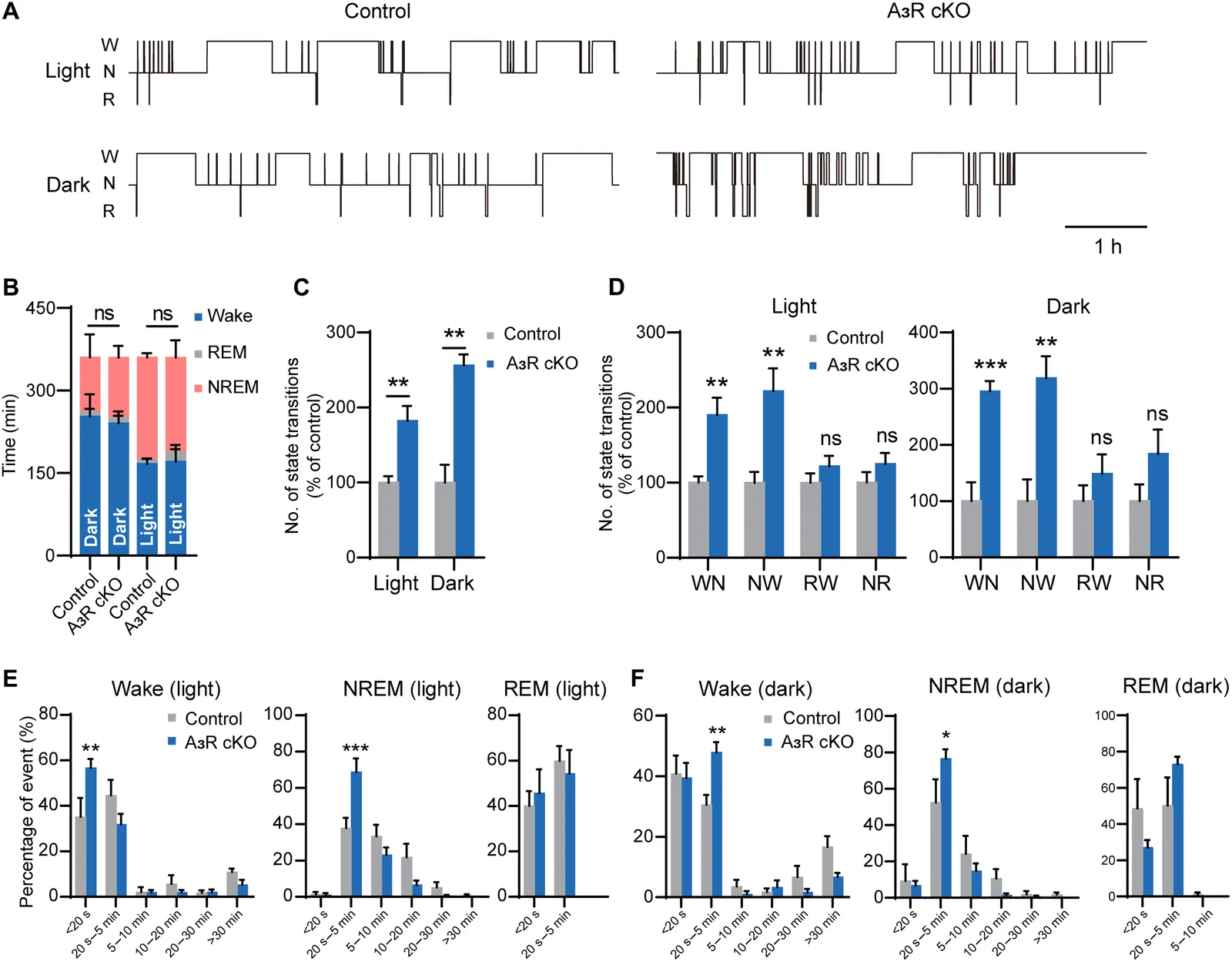
Sleep fragmentation in microglial A_3_R cKO mice. (**A**) A hypnogram measured from microglial A_3_R cKO and sibling control mice at the light phase (ZT3 to ZT9) and dark phase (ZT15 to ZT21). *Cx3cr1^CreERT^::Adora3^fl/fl^* mice, microglial A_3_R cKO mice; *Cx3cr1^WT^::Adora3^fl/fl^* mice, littermate controls. (**B**) Total duration of wakefulness, NREM sleep, and REM sleep at the light phase (ZT3 to ZT9) and dark phase (ZT15 to ZT21) in control and A_3_R cKO mice. *n* = 5 mice per group. Two-way ANOVA with post hoc comparison between control and A_3_R cKO mice. (**C**) Changes in the number of brain state transitions in control and A_3_R cKO mice. *n* = 5 mice per group. Unpaired *t* test was used. (**D**) Number of four brain state transitions in control and A_3_R cKO mice. Unpaired *t* test was used. *n* = 5 mice per group. (**E** and **F**) Percentage of episode duration of wakefulness, NREM sleep, and REM sleep in control and A_3_R cKO mice. Two-way ANOVA with post hoc comparisons test was used. *n* = 5 mice per group. **P < 0.01 and ***P < 0.001. Data are shown as means ± SEM.

To explore the role of microglial A_3_R in sleep regulation, we monitored the sleep-wake patterns in microglial A_3_R cKO mice. Sleep-wake states analysis using EEG/EMG recordings (ZT3 to ZT9 and ZT15 to ZT21) revealed no changes in the total duration of wakefulness, NREM, or REM sleep between control and A_3_R cKO mice (Fig. 4, A and B). However, microglial A_3_R cKO mice exhibited more frequent brain state transitions (Fig. 4, A and C). Further analysis revealed more frequent transitions between wakefulness and NREM sleep (Fig. 4, A and D), as both states were fragmented into shorter episodes (Fig. 4, E and F).

Microglial A_3_R cKO mice exhibited a higher proportion of brief microarousals (<20 s) (*33*) in the light phase (Fig. 4E) and short arousals (20 s to 5 min) in the dark phase (Fig. 4F). The proportion of short NREM sleep also increased during light/dark phases, while REM architecture remained stable (Fig. 4, E and F). These results indicate that microglial A_3_R signaling contributes to maintaining the stability of wakefulness and NREM sleep.

### Microglial A_3_R regulates state-dependent Ca^2+^ activity during natural sleep-wake cycles

To further test the eADO-A_3_R-Ca^2+^ axis of sleep regulation, we next investigated whether microglial A_3_R modulates state-dependent Ca^2+^ dynamics during natural sleep-wake cycles in freely behaving mice. To this end, we generated *Cx3cr1^CreERT^::Adora3^fl/fl^::RCL-GCaMP6s* mice, in which tamoxifen induces microglial-specific knockout of *Adora3* and simultaneous expression of GCaMP6s.

**Fig. 5.**
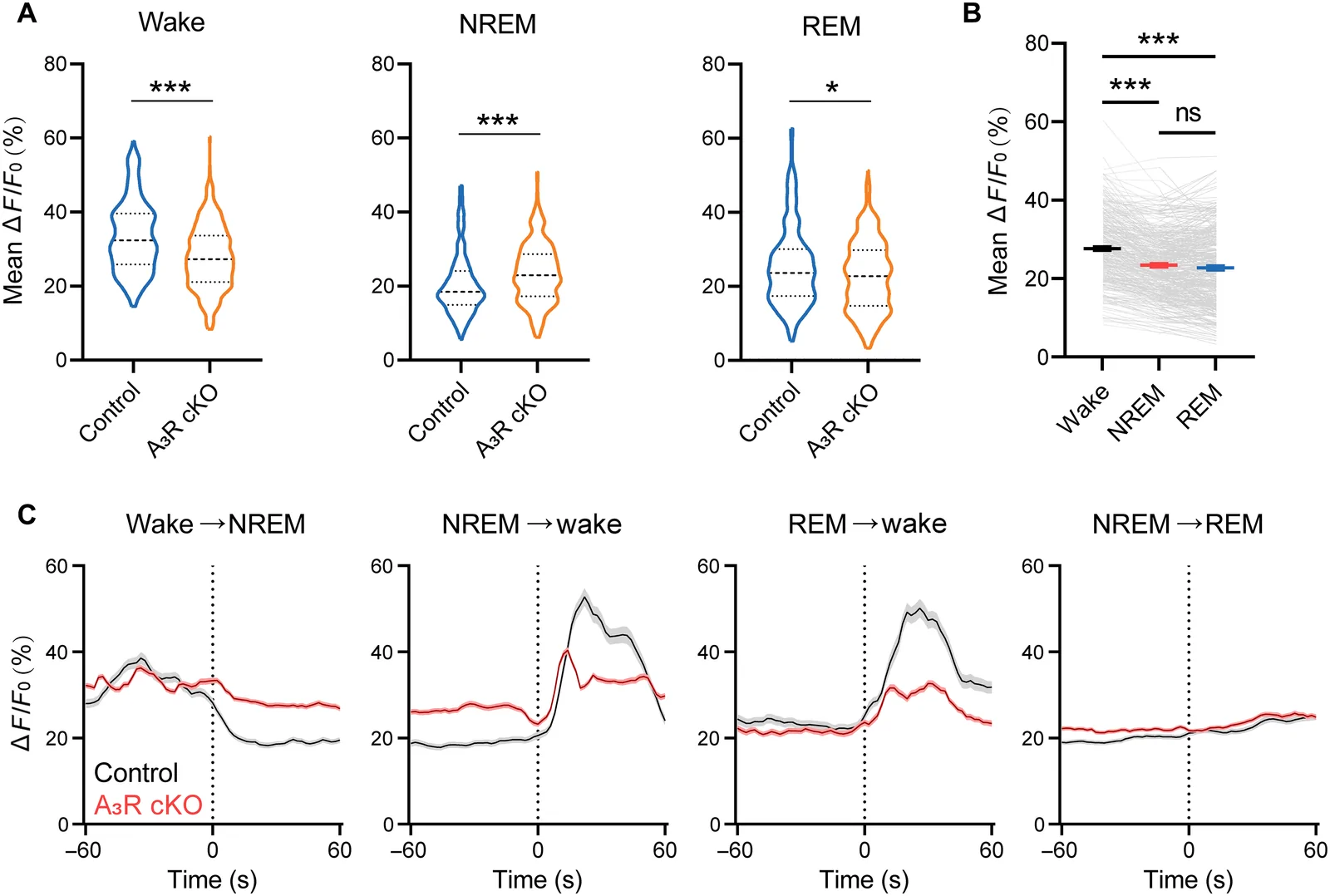
A_3_R regulates state-dependent microglial Ca^2+^ activity. (**A**) Mean Ca^2+^ activity of microglia during different brain states in littermate control and A_3_R cKO mice. Control mice, *Cx3cr1^CreERT^::RCL-GCaMP6s* mice; A_3_R cKO mice, *Cx3cr1^CreERT^::Adora3^fl/fl^::RCL-GCaMP6s* mice. Experimental setup: In vivo imaging was performed 1 month after tamoxifen induction. Mann-Whitney test was used. *n* = 220 cells from 19 sessions of six control mice and *n* = 372 cells from 15 sessions of five A_3_R cKO mice. (**B**) Mean Ca^2+^ activity of microglia in A_3_R cKO mice during different brain states. Data from the same cell are connected by lines. Friedman test with Dunn’s post hoc test was used. *n* = 372 cells from 15 sessions of five mice. (**C**) Ca^2+^ activity during brain state transitions in control and microglial A_3_R cKO mice. The vertical dotted line represents the transition time. *n* = 286, 159, 123, and 328 cells from five control mice. *n* = 620, 540, 360, and 686 cells from five A_3_R cKO mice. *P < 0.05 and ***P < 0.001. Data are shown as means ± SEM.

Compared with *Cx3cr1^CreERT^::RCL-GCaMP6s* control mice, microglial Ca^2+^ activity in A_3_R cKO mice was significantly decreased during wakefulness and REM sleep but increased during NREM sleep (Fig. 5A). Consequently, the differences in microglial Ca^2+^ activity across wakefulness and sleep were attenuated (Fig. 5, A and B). Further analysis showed that, similar to controls, A_3_R cKO mice exhibited higher microglial Ca^2+^ activity during wakefulness than during sleep (Figs. 2D and 5B). However, microglial Ca^2+^ activity did not differ significantly between NREM and REM sleep in A_3_R cKO mice (Fig. 5B).

Although microglial Ca^2+^ activity in A_3_R cKO mice still followed the general trend of responding to brain state transitions, the amplitude of these transitions was significantly reduced (Fig. 5C). Collectively, these findings indicate that microglial A_3_R is essential for maintaining state-dependent Ca^2+^ activity and for amplifying Ca^2+^ dynamics during transitions, thereby enabling microglia to synchronize their activity with ongoing sleep-wake cycles. The attenuation of wake-sleep Ca^2+^ change and the loss of NREM-REM Ca^2+^ difference may underlie the more frequent brain state transitions between wakefulness and NREM sleep.

### A_3_R regulates microglial morphology during natural sleep-wake cycles

Our previous work demonstrated that microglial morphology changes are brain state–dependent and modulated by sleep need (*14*). In cultured primary microglia, A_3_R promoted ADP-induced microglial process extension (*26*), suggesting a role for A_3_R in mediating microglial morphological plasticity. Since microglial morphology is closely linked to intracellular Ca^2+^ signaling, we next asked whether A_3_R also regulates microglial morphology across natural sleep-wake cycles.

**Fig. 6.**
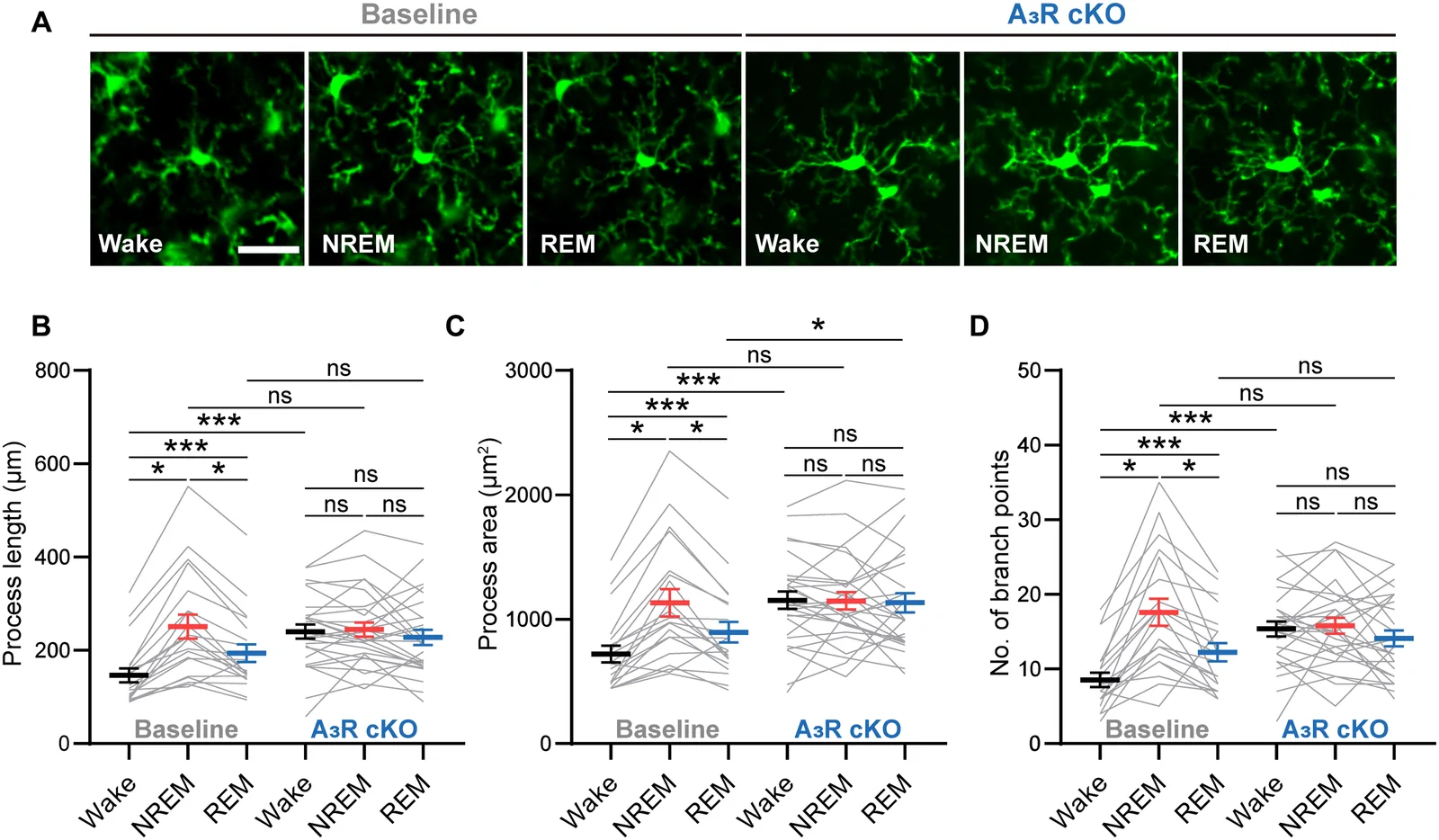
A_3_R alters state-dependent morphological dynamics in microglia. (**A**) Representative microglial morphological changes during the sleep-wake cycle in microglial A_3_R cKO mice (*Cx3cr1^CreERT^::Adora3^fl/fl^::Cx3cr1^GFP/−^* mice). Data collection was performed as follows: Baseline data were acquired at 4 weeks postsurgery, tamoxifen was induced at 5 weeks, and A_3_R cKO data were collected at 9 weeks postsurgery. Scale bar, 30 μm. (**B** to **D**) Quantification of microglial processes length (B), area (C), and number of branch points (D) before (baseline) and after (A_3_R cKO) tamoxifen-induced microglial A_3_R conditional knockout. Data from the same microglia are connected by lines. Friedman test with Dunn’s post hoc test (baseline: length and area) and one-way ANOVA with Tukey’s post hoc test (baseline: branch points; A_3_R cKO: length, area, and branch points) were used. Data are from five mice (baseline, *n* = 20 cells; A_3_R cKO, *n* = 27 cells). *P < 0.05 and ***P < 0.001. Data are shown as means ± SEM.

To address this, we generated *Cx3cr1^CreERT^::Adora3^fl/fl^::Cx3cr1^GFP/−^* mice, in which tamoxifen induces microglial-specific conditional knockout of *Adora3* in green fluorescent protein (GFP)–labeled microglia (Fig. 6A). In control mice without gene knockout, microglial processes exhibited dynamic changes in response to brain state (Fig. 6, A to D). Specifically, microglial process length, territory area, and branch point number were greater during NREM sleep than during wakefulness or REM sleep and lowest during wakefulness (Fig. 6, B to D). This result is consistent with our previous findings (*14*), confirming that microglial morphology is dynamically remodeled by sleep state. However, in microglial A_3_R cKO mice, these morphological differences across brain states were abolished. The length, area, and branch point number of microglial processes during wakefulness were increased compared to those in control mice (Fig. 6, A to D), demonstrating a disruption of normal state–dependent regulation. Together, these results indicate that microglial A_3_R is essential for state-dependent morphological and functional changes in microglia across natural sleep-wake cycles, highlighting the role of adenosine signaling in microglial dynamics.

## DISCUSSION

**Fig. 7.**
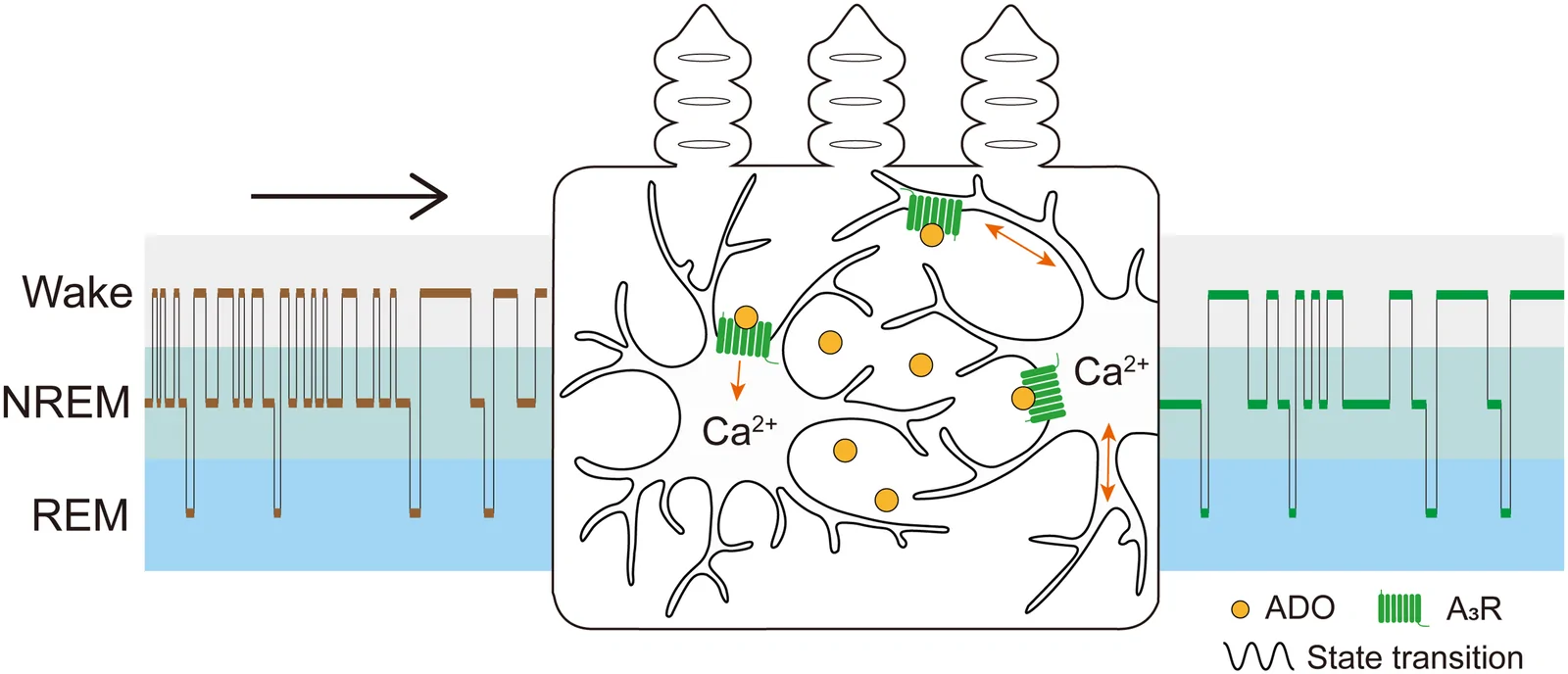
Microglia as stabilizers of sleep homeostasis. Regulation of A_3_R-mediated microglial Ca^2+^ activity and morphology dynamics by eADO oscillations underlies the stabilization of wake-sleep states in mice. Microglia serve as a stabilizer for sleep homeostasis through the eADO-A_3_R signaling pathway.

In this study, leveraging mTPM in freely behaving mice, we delineate the role of microglial eADO-A_3_R-Ca^2+^ signaling axis in sleep homeostasis. We demonstrate that cortical eADO levels and microglial Ca^2+^ activity exhibit brain state–dependent dynamics (Figs. 1 and 2), covarying with sleep pressure (Fig. 1). Critically, we identify microglial A_3_R as the key receptor transducing fluctuating eADO levels into intracellular Ca^2+^ signals and establish that disruption of this axis fragmentizes sleep-wake architecture and abolishes state-dependent microglial morphological plasticity (Fig. 7). These findings collectively define a previously unidentified purinergic-immune pathway wherein microglia, via A_3_R, act as dynamic sensors of sleep pressure to regulate sleep and wakefulness stability.

Adenosine receptors predominantly regulate sleep/wake states by modulating neuronal activity and neurotransmitter systems (*9*). Beyond the well-characterized roles of neuronal adenosine receptors (A_1_R and A_2A_R) in sleep regulation (*34*–*40*), we identify microglial A_3_R as a pivotal nonneuronal mediator of sleep homeostasis. Previous studies showed that constitutive knockout of A_3_R increased nocturnal activity in female mice in the dark and reduced behavioral activation to drug (caffeine and amphetamines) stimuli (*41*). We show that microglial A_3_R maintains state-dependent Ca^2+^ activity and morphological dynamics, thereby curbing transitions between wakefulness and NREM sleep and promoting the stability of both states. Notably, as a G_i_/G_q/11_-coupled protein receptor (*7*), A_3_R induces intracellular Ca^2+^ accumulation and inhibits adenylyl cyclase activity, suggesting that microglial A_3_R may modulate Ca^2+^ activity through coordinated adenosine 3′,5′-monophosphate–dependent signaling to influence sleep-wake states. Given the global microglial A_3_R deletion in this study, the regional specificity of microglial A_3_R function in Ca^2+^ activity and sleep regulation remains unknown. Future region-specific knockout studies are needed to validate the generalizability of our cortical findings.

The disruption of the eADO-A_3_R-Ca^2+^ signaling axis in our study leads to a defined sleep phenotype characterized by fragmentation of wakefulness and NREM sleep, without affecting total sleep duration or REM sleep dynamics (Fig. 4). This “instability” phenotype, marked by increased state transitions and shorter wake/NREM bout durations, coincides with attenuated state-dependent fluctuations in microglial Ca^2+^ activity and morphological plasticity in A_3_R cKO mice (Figs. 5 and 6), highlighting the axis’s role in maintaining sleep-wake stability rather than gross sleep quantity. This phenotype aligns with a growing body of literature indicating that microglia are important for NREM sleep stability and architecture. Global microglial depletion models consistently report disrupted NREM sleep (*15*–*17*), often accompanied by alterations in total NREM sleep time (*15*, *17*)—a more pronounced effect than the specific signaling disruption observed in our A_3_R cKO model. This distinction underscores that while the presence of microglia broadly supports sleep homeostasis, specific signaling pathways, such as the eADO-A_3_R-Ca^2+^ signaling axis, fine-tune particular aspects of state stability. Our findings thus refine the understanding of microglial sleep regulation by revealing an adenosine-mediated pathway through which microglia govern the consolidation of wakefulness and NREM sleep, likely by modulating neural circuit excitability at state transition points.

A critical unresolved issue—and also a limitation of the present study—is how the microglial downstream signaling triggered by the eADO-A_3_R-Ca^2+^ signaling axis coordinates with other neural cells during the sleep-wake cycle. Emerging evidence suggests that NE (*42*, *43*) and purinergic system (particularly ADO and ATP) (*44*) constitute a central hub that integrates glial and neuronal activity (*45*) to regulate sleep (*18*). On one hand, similar to microglia, astrocytes exhibit brain state–dependent Ca^2+^ activity (*43*, *46*–*48*)—a feature highly conserved across species such as mice (*47*, *48*) and *Drosophila* (*46*), with higher Ca^2+^ activity during wakefulness (*47*, *48*). During wakefulness, Ca^2+^-dependent ATP release driven by astrocytes (*49*, *50*), partially regulated by NE (*43*, *45*), may activate microglial P_2_Y_12_-G_i_ signaling, thereby enhancing microglial Ca^2+^ activity (*18*). This G_i_ pathway–mediated enhancement of microglial Ca^2+^ activity (likely via P_2_Y_12_ and A_3_R) further promotes eADO accumulation, which, in turn, suppresses NE release, reduces arousal levels, and facilitates sleep (*18*). In parallel, increased microglial Ca^2+^ activity stimulates the release of cytokines such as TNF-α (*51*), a promoter of sleep (*52*).

On the other hand, microglia metabolize ATP to ADO via CD39 and CD73 (*44*). Elevated eADO inhibits neuronal excitability by activating neuronal A_1_R (*44*), which may promote sleep. Concurrently, adenosine acts on microglial A_3_R to regulate microglial Ca^2+^ activity (Figs. 3 and 5) and surveillance (Fig. 6), which contributes to the maintenance of sleep homeostasis. Together, these processes highlight the synergistic roles of adenosine receptor subtype diversity and cell type specificity in sleep homeostasis regulation. Furthermore, eADO release may exhibit brain region heterogeneity. In the basal forebrain (*49*, *53*) and somatosensory cortex (Fig. 2), eADO displays similar sleep-wake dynamics; however, its dynamics in the parafacial zone differ (*48*), showing an opposite trend particularly during REM sleep (*48*, *49*, *53*). This finding suggests that, in addition to cell type and adenosine receptor subtype, regional differences in local eADO levels may also serve as a key factor in maintaining sleep homeostasis. Future studies should therefore focus on elucidating the roles of differential expression of adenosine receptor subtypes across distinct brain regions and cell types in shaping sleep architecture.

Beyond the potential impact of distinct wakefulness and sleep states on microglial dynamics (Fig. 1), prior studies have elucidated that the attributes of these dynamics vary across both subcellular compartments and brain regions (*18*, *54*, *55*). Throughout the sleep-wake cycle, microglial morphology also exhibits region-specific traits (*55*). For example, in vitro brain slice recordings revealed that somatosensory cortical microglia occupy smaller territories than those in the hippocampus or basal forebrain, with morphological changes related to slow-wave activity intensity (*55*). Consistent with this, our prior findings demonstrated that microglial morphology in the somatosensory cortex is brain state dependent (*14*). Functionally, chemogenetic activation of G_i_ protein–mediated microglial Ca^2+^ activity in the prefrontal cortex or basal forebrain modifies the sleep/wake states, while similar manipulations in the dorsal striatum do not (*18*). This indicates that the influence of microglial Ca^2+^ activity on sleep regulation varies across different brain regions. Moreover, we observed that microglial Ca^2+^ activity peaked during wakefulness in the somatosensory cortex of freely behaving mice (Fig. 1), whereas it was reported to be higher during NREM sleep in the prefrontal cortex of head-fixed mice (*18*). This suggests that microglial Ca^2+^ activity is region specific across the brain during the sleep-wake cycle, and the variations in local neuronal activity may be a key driver of these distinct patterns.

In summary, our study establishes microglial A_3_R as a central mediator of Ca^2+^ dynamics and morphology during natural sleep-wake cycles. By decoding brain state–specific fluctuations in eADO, microglial A_3_R, partly through this receptor, helps consolidate sleep architecture by reducing state transition frequency and preserving stability, thereby contributing to sleep homeostasis. Future studies should elucidate the molecular pathways downstream of A_3_R and investigate how microglia interact with other sleep-regulating cells, including neurons and astrocytes. As demonstrated here, 24-hour continuous in vivo imaging and multimodal recording in freely behaving animals would empower neuroscientists to gain novel insights into these important questions.

## MATERIALS AND METHODS

### Animals

All experimental procedures were conducted following the approval of the Institutional Animal Care and Use Committee of PKU–Nanjing Institute of Translational Medicine (approval ID: IACUC-2021-023). C57BL/6 mice aged 2 to 3 months were used in most of the experiments, with a few longer data collection periods using mice at around 4 months of age. Mice were housed with a standard 12-hour light/12-hour dark cycle and fed standard chow ad libitum.

*Cx3cr1^CreERT^* (#020940) mice and *Cx3cr1^GFP/GFP^* (#005582) mice were purchased from the Jackson Laboratory. *Adora3^fl/fl^* (#T006284) mice were purchased from the GemPharmatech Co. Ltd. *RCL-GCaMP6s* mice were provided by Nanjing Raygenitm Biotech Co. Ltd. *Cx3cr1^CreERT^* mice and *Adora3^fl/fl^* mice were crossed to generate the conditional knockout mouse line.

To generate mice with microglia-specific expression of GCaMP6s and A_3_R knockout, we bred *RCL-GCaMP6s::Adora3^fl/−^* mice with *Cx3cr1^CreERT^::Adora3^fl/−^* mice to obtain *Cx3cr1^CreERT^::RCL-GCaMP6s::Adora3^fl/fl^* mice. Similarly, to generate mice with microglia-specific expression of GFP and A_3_R knockout, we bred *Cx3cr1^GFP/GFP^::Adora3^fl/−^* mice with *Cx3cr1^CreERT^::Adora3^fl/−^* mice to obtain *Cx3cr1^CreERT^::Cx3cr1^GFP/−^::Adora3^fl/fl^* mice. To activate tamoxifen-induced CreERT, mice were intraperitoneally injected at ∼8 weeks of age with 100 μl of tamoxifen (20 mg/ml; T5648, Sigma-Aldrich) for 5 consecutive days, and considering the recovery period of cranial window surgery and the macrophage turnover, mice that had been induced with tamoxifen 1 month prior were selected for data acquisition.

### Surgery

All surgical interventions were performed aseptically, with all substances administered to the animals being sterile. The mice were anesthetized using isoflurane (1.5% in air at a flow rate of 0.5 liter/min) and kept on a 37°C heating pad throughout the surgery. The region of the cerebral cortex to be imaged was identified on the basis of stereotactic coordinates (somatosensory area, from bregma: anteroposterior, −1 mm; mediolateral, +2 mm). A 4-mm cranial window was created using a high-speed microdrill under the guidance of an anatomical microscope. Glass coverslips were implanted and fixed onto the somatosensory cortex. Mice were used after 4 weeks of postoperative recovery.

Implantation of epidural screw electrodes (with a diameter of 0.8 mm) was performed at opposite sides of the imaging window to enable continuous EEG recording from the frontal cortex (bregma: anteroposterior, +1.5 mm; mediolateral, +1.5 mm) and parietal cortex (anteroposterior, −2 mm; mediolateral, +2.5 mm). The electrodes were fixed to the skull using dental cement. In addition, another set of electrodes was inserted into the neck muscles for EMG recording. Subsequently, a headpiece baseplate was affixed to the skull using cyanoacrylate and reinforced with dental cement. Mice were used after at least 2 weeks of postoperative recovery.

### Virus injection

AAV9-hsyn-GRAB_ADO1.0_ (200 nl; PT-1348, BrainVTA) was injected into the somatosensory cortex (anteroposterior, −1 mm; mediolateral, +2 mm; dorsoventral, −0.3 mm) at a rate of 20 nl/min. A 4-mm-diameter glass coverslip was implanted and fixed on the somatosensory cortex. The titer of the ADO sensor viruses was estimated to be ≥2 × 10^12^ vg/ml. The mice were used 1 month after postoperative recovery.

### Two-photon imaging and sleep recording

Mice recovered from surgery without cranial window infection and with typical EEG/EMG signals were selected for imaging. Initial imaging stacks were acquired to select the best FOV using mTPM (Transcend Vivoscope) on head-fixed mice (*27*, *28*). For Ca^2+^ activity imaging, suitable FOVs with rich Ca^2+^ activity of microglial processes were selected at a depth of 50 to 100 μm below the dura mater. For microglial morphology imaging, appropriate FOVs containing 5 to 10 microglial somata were selected. To achieve multiday imaging of the same FOV and repeated mounting, the mTPM holder was sealed onto the headpiece baseplate positioned above the coverslip.

FHIRM-TPM was used to collect imaging data (*27*, *28*). Imaging of Ca^2+^ activity and morphology was performed in the high-resolution model [lens numerical aperture (NA), 0.7; FOV, 220 μm by 220 μm; lateral resolution, 0.74 μm; axial resolution, 6.53 μm] combined with ETL model (*27*, *28*). The sampling frame rate for Ca^2+^ activity and morphology imaging was 10 to 14 Hz and 5 to 7 Hz, respectively. In addition, large FOV model (lens NA, 0.75; FOV, 420 μm by 420 μm; lateral resolution, 1.13 μm; axial resolution, 12.2 μm) was used in adenosine imaging. The sampling frame rate was 5 Hz.

The continuous and simultaneous recording of EEG/EMG signals was performed using the Vital Record system developed by Kissei Comtec, while mouse behavior was monitored with an infrared video camera. Mice used for imaging and recording purposes were housed in the behavioral area for 3 days before the experiment to allow acclimation. During this period, they were allowed unrestricted access to food and water.

### Drug application in vivo

A drug infusion catheter filled with ACSF was placed in the subarachnoid space adjacent to the imaging area of the cranial window. The catheter and the skull were held together using biological tissue glue and dental cement, and then the headpiece baseplate was affixed to the skull using cyanoacrylate and reinforced with dental cement. Mice were used after at least 2 weeks of postoperative recovery. During the administration process, the needle of the microsampler was positioned deep within the catheter and close to the opposite end of the catheter. A total volume of 10 μl of solution was delivered through the catheter and into the brain. Drugs were applied at the following concentrations: 500 nM adenosine (ST1075, Beyotime Biotechnology), 300 nM Cl-IB-MECA (C277, Sigma-Aldrich), 1 μM MRS1523 (M1809, Sigma-Aldrich), 1 μM *N*^6^-cyclohexyladenosine (HY-18939, MCE), 1 μM regadenoson (HY-A0168, MCE), 300 nM BAY 60-6583 (HY-103171, MCE), 200 nM DPCPX (HY-100937, MCE), 200 nM vipadenant (HY-10857, MCE), 200 nM MRS 1754 (HY-14121, MCE) or ACSF. Microglial Ca^2+^ activity was recorded before and 30 min after administration.

### Sleep deprivation

Mice were deprived of sleep for 6 hours using a sleep deprivation device (XR-XS108, Shanghai Xinsoft Information Technology). The parameters were set as follows: running direction of the bar, positive and negative rotation; stripping strength, moderate; rotating speed, 5 rpm; running mode, random.

### Microglial isolation from adult mice

Cortical microglia were isolated from A_3_R cKO and control mice 1 month after tamoxifen induction. Anesthetized mice were perfused with prechilled phosphate-buffered saline (PBS) to obtain brain samples. The cerebral cortex was dissected, thoroughly chopped, and then digested with 200 UI of papain (P3125, Sigma-Aldrich) at 37°C for 30 min, followed by termination of the digestion with a medium containing 10% fetal bovine serum (18045088, Gibco). The solution was filtered through a 70-μm cell strainer to remove debris. The cell suspension was centrifuged at 300*g* for 5 min, and the cell pellets were resuspended in 4 ml of 30% Percoll plus (P331004, Aladdin), transferred to a 15-ml centrifuge tube (CSP020015, Jet Biofil) containing 4 ml of 70% Percoll plus, on which 2 ml of PBS was layered, and then centrifuged at 18°C for 40 min, and the top layer of myelin was removed. The cell pellets were washed twice with fluorescence-activated cell sorting (FACS) buffer, and CD11b (101211, BioLegend) and CD45 (157213, BioLegend) immunostaining was performed on ice. Microglia and negative cells were sorted using a flow cytometer (FACSAria II, Becton Dickinson). After FACS, the A_3_R expression level was detected by mRNA isolation and real-time quantitative PCR.

### Quantitative real-time PCR

Total RNA was extracted from primary cells enriched by centrifugation, according to the guidelines of the RaPure cell RNA kit (R4010, Magen). First-strand cDNA was synthesized using the M-MLV Reverse Transcriptase kit (R223, Vazyme). Quantitative real-time PCR was conducted using a Hieff qPCR SYBR Green Master Mix (No Rox) kit (11201ES03, Yeasen). The mRNA level of β*-actin* was analyzed as an internal control. The following primer sequences were used: *Adora3*, ACATGCTGGTCATCTGGGTG (forward) and AGCAGCACACAGGACATGAA (reverse); β*-actin*, TTGGGTATGGAATCCTGTGGC (forward) and GCTAGGAGCCAGAGCAGTAATC (reverse).

### Immunofluorescence

Brain slices fixed with 4% paraformaldehyde were permeabilized by treating with 0.5% Triton X-100. Following a 2-hour blocking period in PBS containing 2% bovine serum albumin at 37°C, brain slices were incubated overnight at 4°C with rabbit-derived primary antibody against IBA1 (17198, Cell Signaling Technology), glial fibrillary acidic protein (80788, Cell Signaling Technology), or NeuN (24307S, Cell Signaling Technology). Slices were then incubated in the dark with a fluorescently labeled secondary antibody for 2 hours at 37°C and washed with PBS. Last, slices were sealed with an antifluorescent quench agent containing 4′,6-diamidino-2-phenylindole.

### Quantitative analysis

#### Microglial Ca^2+^ activity analysis

The time-lapse stack was aligned offline using the Template-Matching plugin of ImageJ Fiji software to correct for *xy* motion artifacts. To analyze the changes of long-term Ca^2+^ activity in microglial processes in freely behaving mice, ImageJ Fiji was used to delineate the region of interest (ROI) and extract the data, which were then processed in MATLAB (MathWorks). Raw data were binned into 1 Hz, followed by background autofluorescence subtraction. Δ*F*/*F*_0_ was subsequently calculated, using the fluorescence signal itself. *F*_0_ was defined as the average value of the lower 25th quartile fluorescence signal within each 60-s window of the recorded fluorescence signal (*54*).

In the experiment of drug application (Fig. 3 and figs. S3 and S4), since drug stimulation accelerates the movement of microglial processes, we used AQuA software (*56*), using spatially unfixed and size-varying ROIs, to quantify Ca^2+^ activity in the FOV more accurately. Microglial Ca^2+^ events were identified on the basis of experimentally defined parameters (*47*), which were applied to all motion-corrected images (intensity threshold scaling factor, 2; smoothing, 1; minimum size, 10; temporal cut threshold, 2; growing *z*-threshold, 1; rising time uncertainty, 2; slowest delay in propagation, 2; propagation smoothness, 1; *z*-score threshold, 2).

#### Microglial morphological analysis

The time-lapse stack was aligned offline using the Template-Matching plugin of ImageJ Fiji to correct for *xy* motion artifacts. The image stack of 30 s in different brain states was compressed into one frame to analyze the morphological characteristics of microglia. Subsequently, the microglial morphology was reconstructed and quantified using Imaris 9.5 software. The definitions of microglial process length, area, and branch points remain consistent with our previously description (*14*).

#### Adenosine signal analysis

Raw data were binned into 1 Hz, followed by background autofluorescence subtraction. Δ*F*/*F*_0_ was subsequently calculated, using the fluorescence signal itself. *F*_0_ was defined as the average value of the lower 25th quartile fluorescence signal within each 60-s window of the recorded fluorescence signal. To enhance comparability across different brain states, wake and NREM states with a duration exceeding 30 s were selectively chosen for quantifying eADO signal changes. Mean Δ*F*/*F*_0_ of eADO signals in different states within one session was used for comparison. Microglial Ca^2+^ and eADO signals were represented as *z*-score in representative long-term recordings (Figs. 1B and 2B). To quantify the ADO signal during brain state transitions, the Δ*F*/*F*_0_ was further normalized (Fig. 2E) (*53*).

#### EEG/EMG data analysis

All signals were digitally sampled at a rate of 128 Hz. EEG and EMG signals were processed using bandpass filtering (EEG, 0.1 to 35 Hz; EMG, 10 to 100 Hz). Raw EMG signal passed through a 50-Hz digital notch filter. Signals were converted using fast Fourier transform (point, 256). EEG and EMG recordings were analyzed to classify brain states by the SleepSign software (Kissei Comtec). Each 5-s epoch was assigned as wakefulness, NREM sleep, or REM sleep based on combined EEG and EMG features. Wakefulness was characterized by desynchronized EEG activity alongside high EMG tonus. NREM sleep was identified by synchronized EEG with low-frequency (0.5 to 4 Hz), high-amplitude oscillations and reduced EMG activity. REM sleep was defined by dominant theta-band power (4 to 8 Hz) accompanied by low EMG activity. Consecutive epochs sharing the same state were merged into a single episode. Microarousal, characterized by a desynchronized EEG accompanied by an activated EMG, was defined as brief arousal-like bouts lasting less than 20 s during NREM sleep (*33*). At the same time, combined with behavioral videos, artificial correction was carried out for the states with obvious misjudgments in individual epochs.

In Figs. 1E and 2C, microglial Ca^2+^ and eADO signals were processed using Δ*F*/*F*_0_ normalization before subsequent correlation analyses. Specifically, microglial Ca^2+^ and eADO events were detected using the findpeaks algorithm (threshold, 1.5 Δ*F*/*F*_0_) in MATLAB. After the root mean square conversion of EMG signals, EMG data corresponding to Ca^2+^ events and ADO events were extracted. The area under the curve (AUC) was calculated (*48*, *53*) using trapezoidal integration after baseline [the mean signal during the 100 s preceding the event (*53*)] subtraction. Pearson’s correlation was then performed on the paired AUC values to assess the relationship between event-integrated signal intensities of the two modalities.

In fig. S1B, brain state episode durations were extracted and summarized as mean and median values. Duration distributions were visualized using histograms (bin width, 10 s).

In fig. S2B, to assess the temporal relationship between microglial Ca^2+^ activity and EEG power spectra across different frequency bands, EEG signals were segmented into 2-s nonoverlapping windows, and power spectral density was estimated for each segment using Welch’s method (1-s Hamming window) via MATLAB’s pwelch function (*57*). Absolute power was calculated for delta (1 to 4 Hz), theta (4 to 8 Hz), sigma (8 to 15 Hz), and gamma (30 to 80 Hz) bands and then converted to dB scale by normalizing to the maximum power of each band per session and applying 10 × log_10_ transformation. For each microglial Ca^2+^ peak detected using MATLAB’s findpeaks function (threshold, 1.5 Δ*F*/*F*_0_), a 41-s time window (20 s before and after the peak) was extracted from both the Ca^2+^ signal and each EEG power band signal. All retained events were then individually normalized using *z*-score transformation to enable cross-event comparisons.

To generate fig. S2C, we detected and quantified microglial Ca^2+^ events from Δ*F*/*F*_0_ signals using MATLAB’s findpeaks function with a threshold of 1.5 Δ*F*/*F*_0_. Events were only included if they occurred entirely within stable brain state episodes lasting at least 60 s, as defined by consecutive EEG/EMG-based state labels (wakefulness, NREM, or REM sleep). For each detected event, the onset and offset boundaries were determined using the half-height method, where the start was defined as the time point at which the signal rose above a threshold set at 10% of the peak prominence above the preceding trough and the end was defined as the time point when the signal decayed back to the same level. The rise time was calculated as the interval from event onset to 90% peak, the decay time was calculated as the interval from 90% peak to event offset, and peak amplitude was calculated as the prominence of the event (peak height relative to the preceding baseline trough).

#### Behavioral analysis

Behavioral videos were used to differentiate behaviors such as locomotion, quiet wakefulness, and grooming. Locomotion was characterized by horizontal movement. Quiet wakefulness was defined as a behavioral state in which the mouse remains seated or prone in a stationary position, displaying no large-scale body movements, and was simultaneously determined, via EEG/EMG criteria, not to have entered sleep. Grooming was characterized by stereotyped cephalocaudal self-care actions exhibited when mice stay still, covering forepaw facial cleaning and oral licking over the torso, hind limbs, tail, and genitals. Single scratches lasting under 1 s were discarded from total grooming time, and grooming segments split by pauses ≤ 5 s were counted as one complete bout. Sleep was ascertained through EEG and EMG recordings, including NREM and REM sleep.

In fig. S2A, to examine the relationship between spontaneous behaviors and microglial Ca^2+^ activity, we segmented the continuous microglial Ca^2+^ imaging data into epochs centered at the onset of each behavioral episode. Behavioral states, including locomotion, quiet wakefulness, grooming, and sleep, were classified on the basis of synchronized video recordings (movie S1) or EEG/EMG signals. Only behavioral episodes lasting at least 10 s were included in the analysis to ensure stable state identification. For each valid episode, we extracted a 60-s window (−30 to +30 s relative to the behavior onset) from the Ca^2+^ traces of all recorded microglia.

### Statistical analyses

All statistical analyses were conducted using GraphPad Prism 8 (GraphPad Software). For all tests, a two-tailed approach was used to assess significance. Significance levels were denoted as follow: *P < 0.05, **P < 0.01, and ***P < 0.001. Normality tests were performed on all datasets before further analysis. To determine the significance of differences between groups, appropriate parametric or nonparametric tests were used on the basis of the data distribution and experimental design. Statistical details are provided in the figures and figure legends. All data are presented as the means ± SEM.
