# Supplementary material for: Microglia stabilize sleep homeostasis via adenosine A3 receptor signaling
Source: Sci Adv. 2026 Sep 11;12(37):eaef3957. doi: 10.1126/sciadv.aef3957 (PMC13564824; doi:10.1126/sciadv.aef3957)
Supplement: Supplementary file 1 [file sciadv.aef3957_sm.pdf]

Science Advances
AAAS

Supplementary Materials for

Microglia stabilize sleep homeostasis via adenosine A_3 receptor signaling

Zhong Zhao *et al.*

Corresponding author: Xiaochun Gu, xiaochun.gu@seu.edu.cn; Heping Cheng, chengp@pku.edu.cn

Sci. Adv. **12**, eaef3957 (2026)
DOI: 10.1126/sciadv.aef3957

The PDF file includes:

Figs. S1 to S4
Legend for movie S1

Other Supplementary Material for this manuscript includes the following:

Movie S1

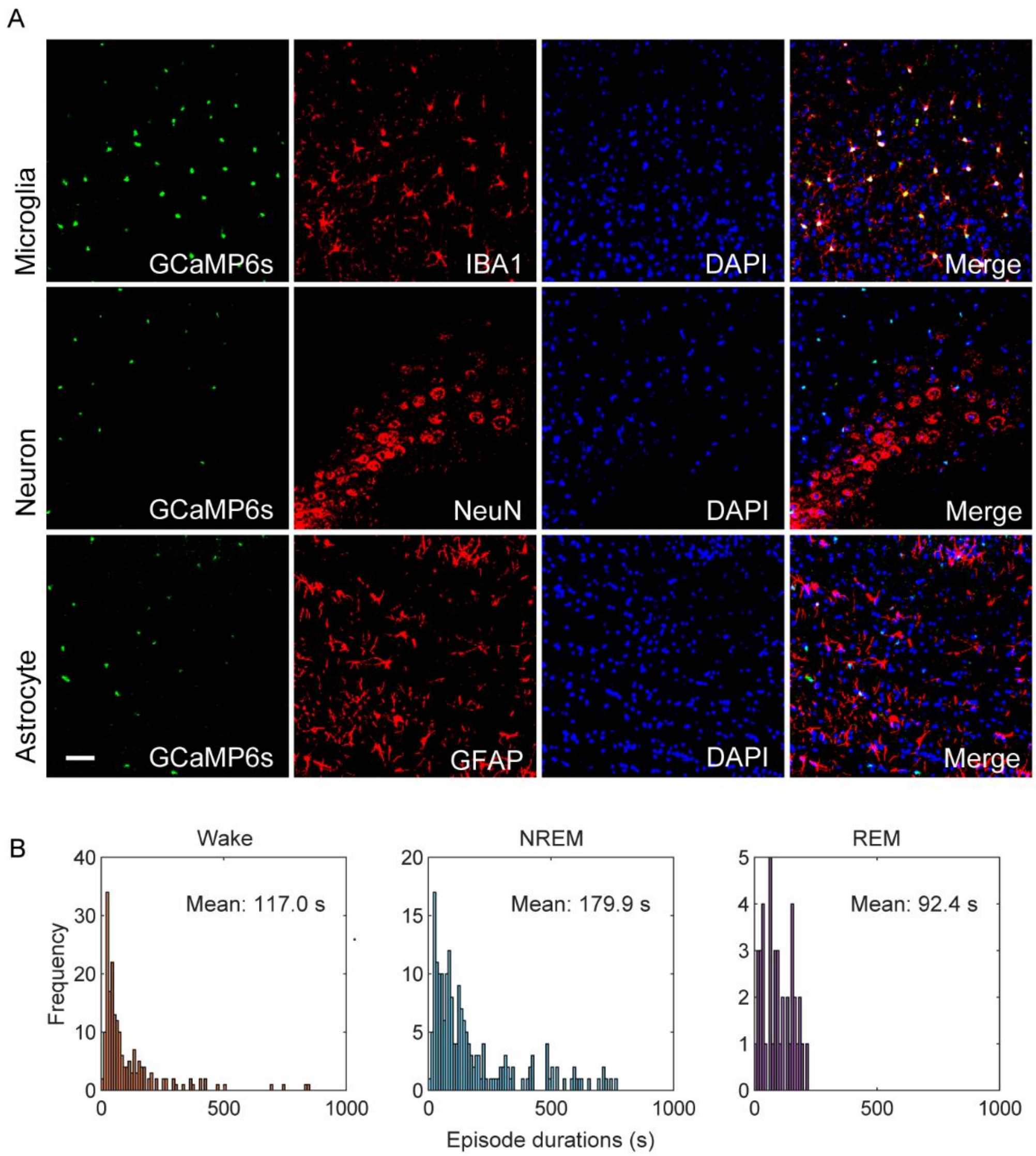
A
Microglia
Neuron
Astrocyte
GCaMP6s
IBA1
NeuN
GFAP
DAPI
Merge
B
Wake
NREM
REM
Mean: 117.0 s
Mean: 179.9 s
Mean: 92.4 s
Frequency
Episode durations (s)
0
500
1000

Fig. S1. Long-term microglial Ca^{2+} imaging in freely behaving mice.
(A) Immunohistochemical verification of GCaMP6s expression in microglia (IBA1), but not in neurons (NeuN) or astrocytes (GFAP). Nuclei were stained with DAPI. Scale bar, 200 µm. **(B)** Distribution of brain state episode durations. The figure displays duration distributions for wakefulness, NREM, and REM sleep across 6 mice. Histograms shows the frequency distribution of segment durations (bin width: 10 s). The mean duration is indicated within each panel.

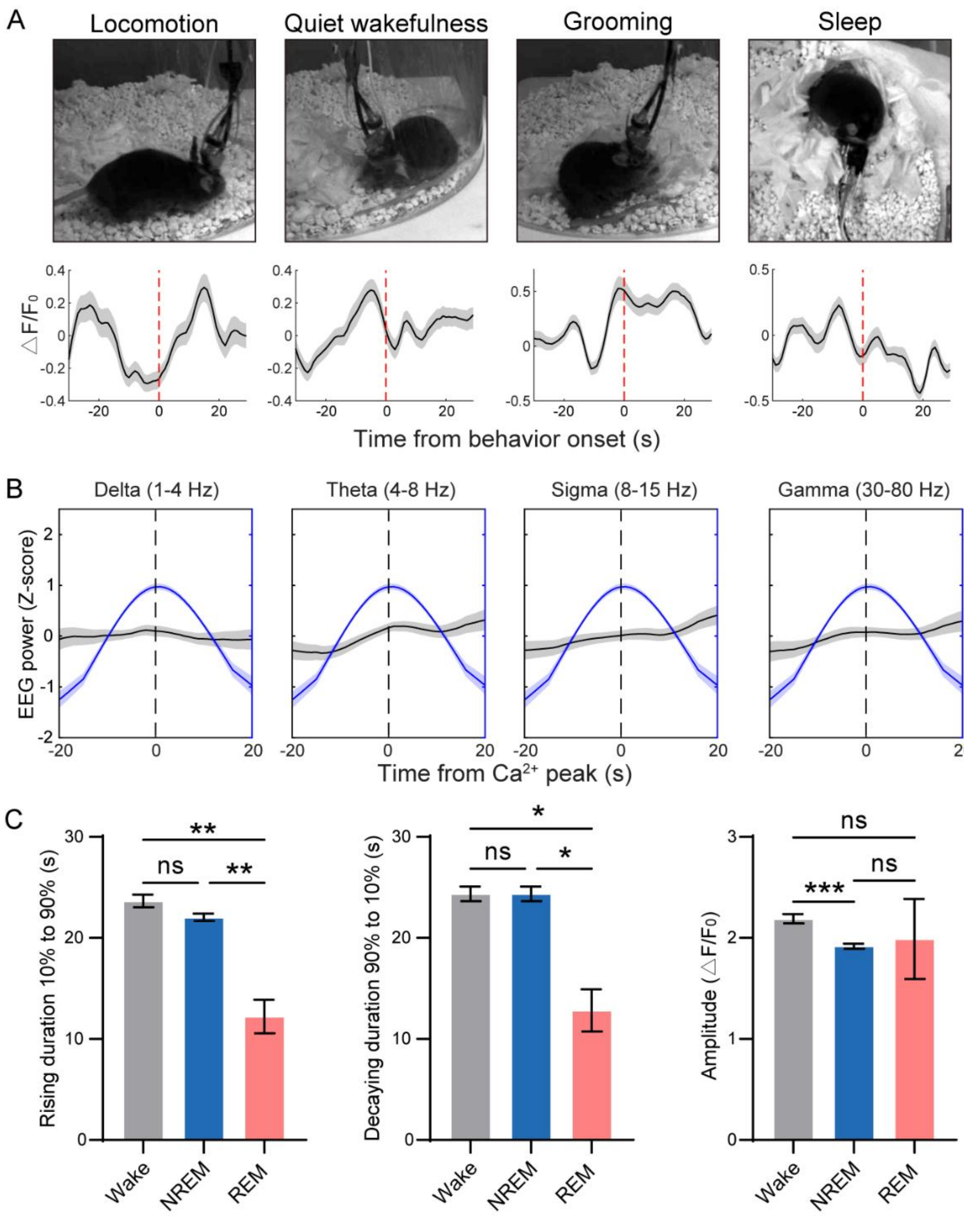
A
Locomotion
Quiet wakefulness
Grooming
Sleep
△F/F0
Time from behavior onset (s)
B
Delta (1-4 Hz)
Theta (4-8 Hz)
Sigma (8-15 Hz)
Gamma (30-80 Hz)
EEG power (Z-score)
Calcium signal (Z-score)
Time from Ca2+ peak (s)
C
Rising duration 10% to 90% (s)
Decaying duration 90% to 10% (s)
Amplitude (△F/F0)
Wake
NREM
REM
ns
**
*
***

Fig. S2. Behavioral and EEG spectral correlates of microglial Ca^{2+} activity in freely moving mice.

(A) Time course of microglial Ca^{2+} activity across locomotion, quiet wakefulness, grooming, and sleep in freely behaving mice. All behavioral episodes were ≥10 s in duration, and microglial Ca^{2+} activity was extracted from a 60-s window. Top, representative images of behavioral states; bottom, *n* = 191, 348, 176, and 221 cells from 9, 20, 11, and 14 behavioral episodes (locomotion, quiet wakefulness, grooming, and sleep, respectively), obtained from 4 mice. **(B)** Temporal relationship between microglial Ca^{2+} events and EEG power spectra. x = 0 denotes the peak of each individual Ca^{2+} event (dashed vertical line). Shown are the mean ± s.e.m. (shaded areas) of z-scored microglial Ca^{2+} signals (blue, left Y-axis) and z-scored EEG power (black, right Y-axis) for four frequency bands: delta (1–4 Hz), theta (4–8 Hz), sigma (8–15 Hz), and gamma (30–80 Hz). *n* = 53 from 4 mice. **(C)** Quantification of the rising duration, decaying duration, and amplitude of microglial Ca^{2+} events during wakefulness, NREM sleep, and REM sleep. *n* = 651, 1553, 11 events from 6 sessions obtained from 5 mice. $^*P < 0.05$, $^{**}P < 0.01$. ns, not significant. Data are shown as mean ± s.e.m.

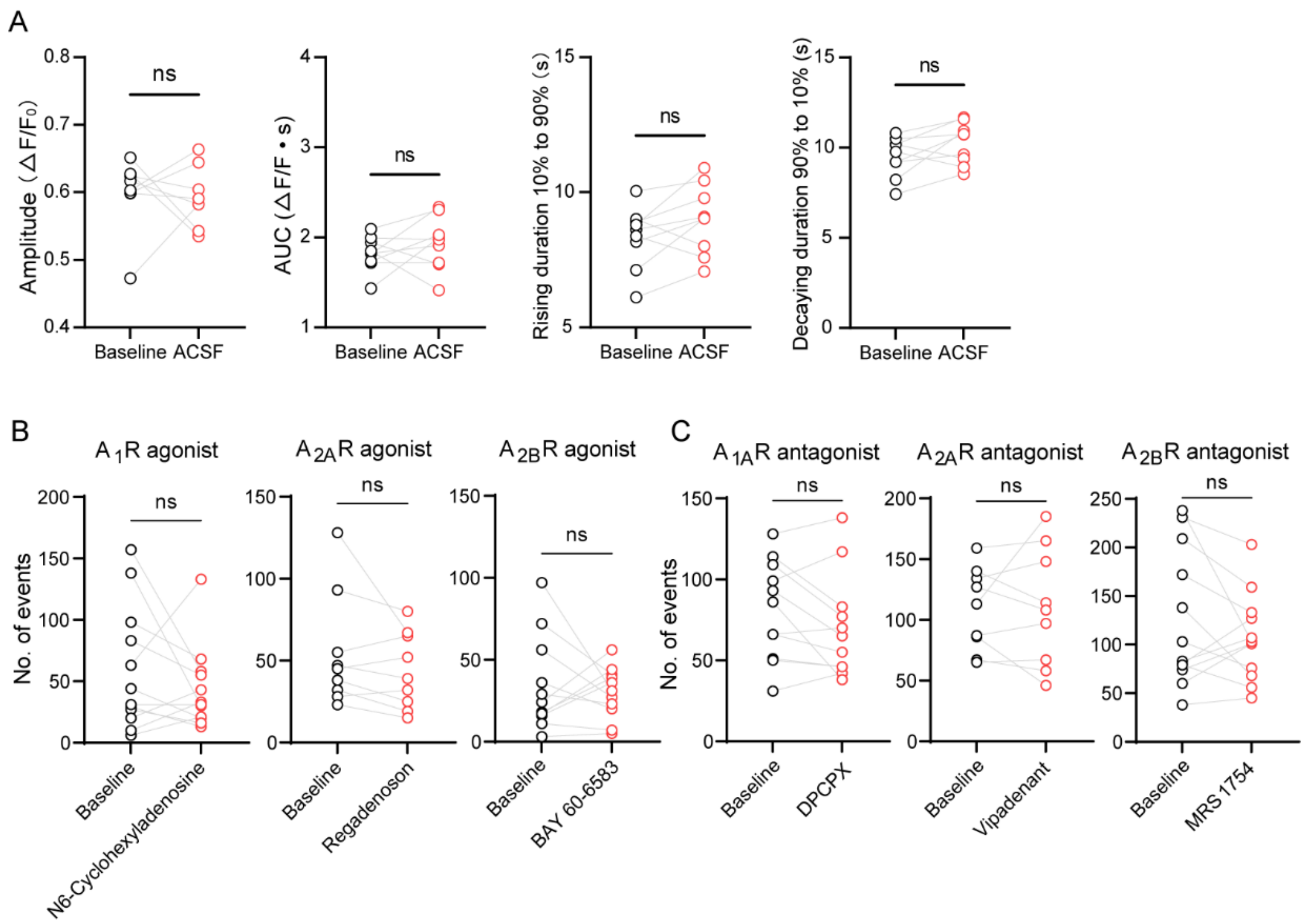

A
Amplitude (ΔF/F0)
AUC (ΔF/F • s)
Rising duration 10% to 90% (s)
Decaying duration 90% to 10% (s)
ns
Baseline ACSF
B
A1R agonist
A2AR agonist
A2BR agonist
No. of events
Baseline
N6-Cyclohexyladenosine
Regadenoson
BAY 60-6583
C
A1AR antagonist
A2AR antagonist
A2BR antagonist
DPCPX
Vipadenant
MRS1754

Fig. S3. Pharmacological modulation of microglial Ca^{2+} activity by adenosine receptor agonists and antagonists.

(A) The amplitude, area under the curve (AUC), and duration of Ca^{2+} activity during microglia processes in the field of view (FOV) were analyzed in 5-minute recordings 30 min after ACSF administration. Data from the same FOV are connected by lines. *n* = 9 cells from 3 mice. Wilcoxon test (event, amplitude, AUC, rising duration, and decaying duration), two-tailed paired t test (duration). **(B, C)** The number of Ca^{2+} events within the FOV was analyzed in 5-minute recordings, 30 min after agonist (B) or antagonist (C) administration of A_1R, $A_{2A}R$, and $A_{2B}R$. Data from the same FOV are connected by lines. *n* = 9 FOVs from 3 mice (group, $A_{2A}R$ agonist; group, $A_{2A}R$ antagonist). *n* = 11 FOVs from 3 mice (group, $A_{2B}R$ agonist; group, A_1R antagonist). *n* = 12 FOVs from 3 mice (group, A_1R agonist; group, $A_{2B}R$ antagonist). Wilcoxon test (group, A_1R agonist; group, $A_{2A}R$ agonist), two-tailed paired t test (group, $A_{2B}R$ agonist; group, A_1R antagonist; group, $A_{2A}R$ antagonist; group, $A_{2B}R$ antagonist). ns, not significant.

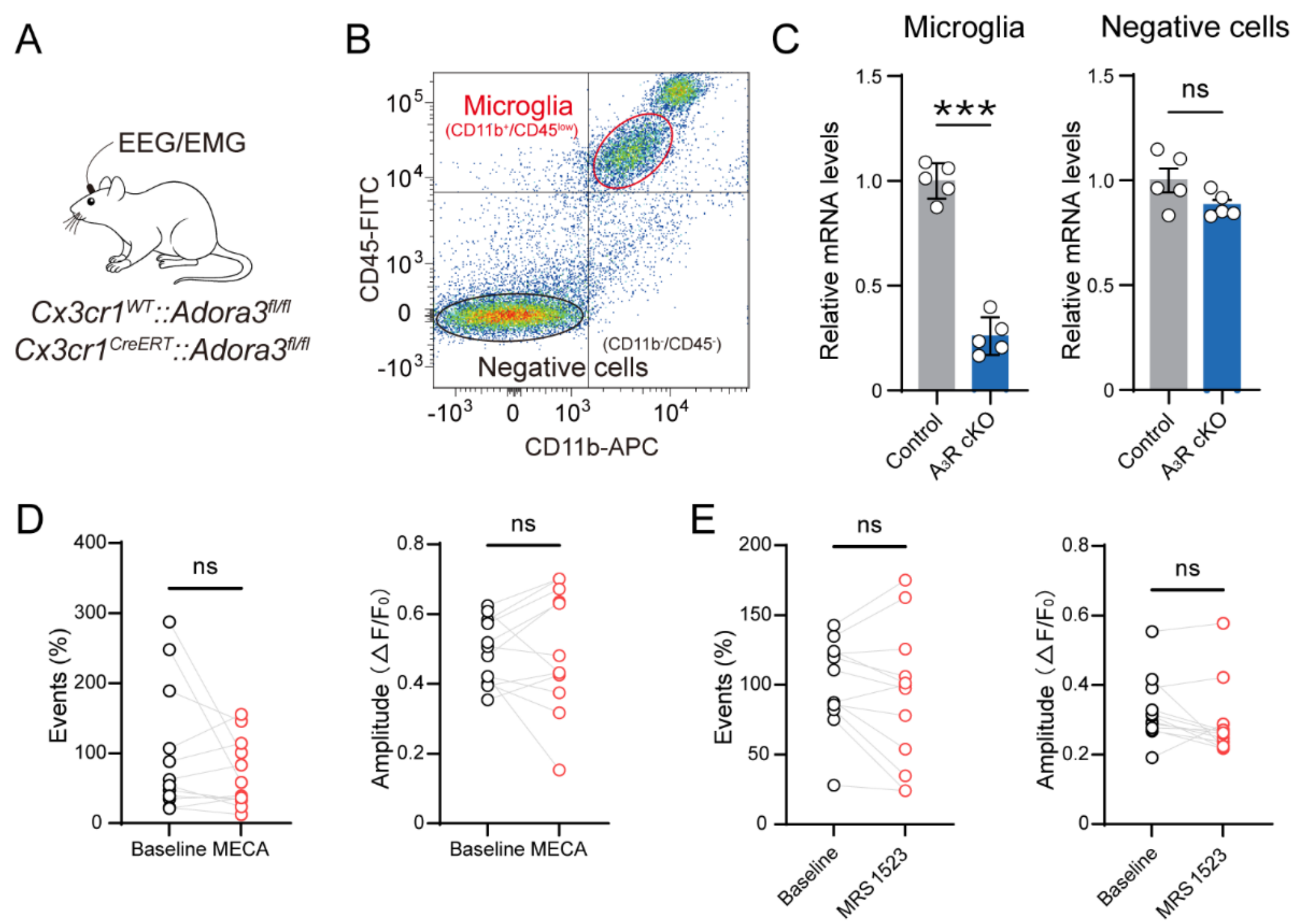

A
EEG/EMG
Cx3cr1WT::Adora3fl/fl
Cx3cr1CreERT::Adora3fl/fl
B
Microglia
(CD11b+/CD45low)
CD45-FITC
(CD11b-/CD45-)
Negative cells
CD11b-APC
C
Microglia
Negative cells
Relative mRNA levels
***
ns
Control
A3R cKO
D
Events (%)
ns
Baseline MECA
Amplitude (ΔF/F0)
E
Baseline
MRS 1523

Fig. S4. A_3R regulates microglial Ca^{2+} activity in microglia-specific A_3R conditional knockout mice.

(A) Experimental paradigm: cortical microglia were isolated one month after tamoxifen induction. *$Cx3cr1^{WT}$::$Adora3^{fl/fl}$* mice served as littermate controls. **(B)** $CD11b^+/CD45^{low}$ cells (microglia) and $CD11b^-/CD45^-$ (negative cells) were collected by FACS. **(C)** Quantitative RT-PCR showing reduced A_3R mRNA expression in microglia from A_3R cKO mice relative to control mice. Unpaired t test was used. $n = 5$ mice per group. **(D-E)** The number of events and the amplitude of Ca^{2+} activity during microglial processes in the FOV were analyzed in 5-minute recordings 30 min after Cl-IB-MECA (D) or MRS1523 (E) administration in microglial A_3R cKO mice. $n = 12$ FOVs from 3 mice. Data from the same FOV are connected by lines. Two-tailed paired t test (D; E, events), wilcoxon test (E, amplitude). $^{***}P < 0.001$. ns, not significant. Data are shown as mean ± s.e.m.

Movie S1. Simultaneous behavioral video recordings of mice during *in vivo* imaging.

Representative top-view and side-view synchronized recordings of a freely behaving mouse during long-term *in vivo* imaging. Four spontaneous behavioral states were presented: locomotion, quiet wakefulness, grooming, and sleep. The mouse was equipped with a head-mounted mTPM and an EEG/EMG recording module.
